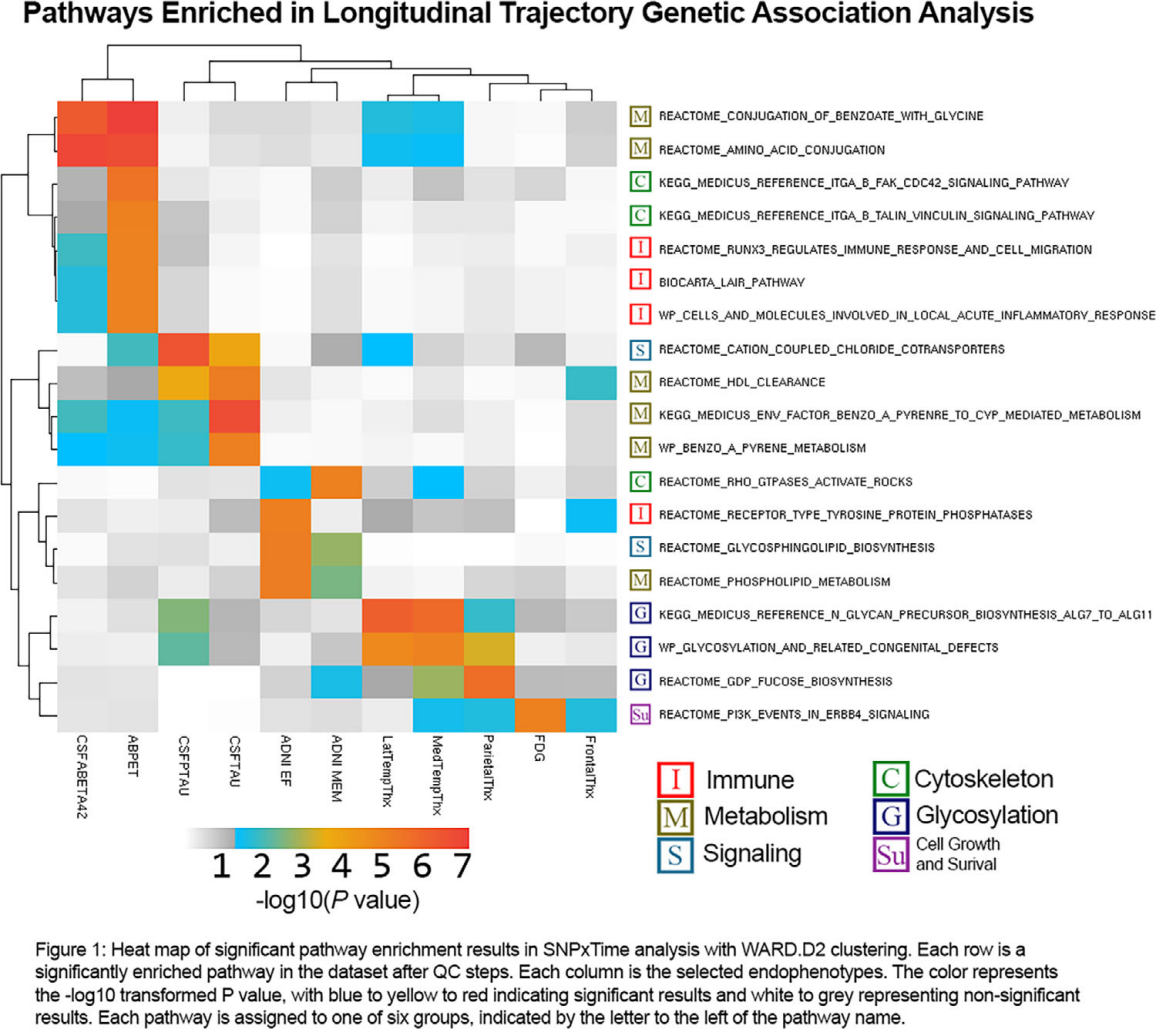# Pathway Enrichment of Longitudinal AD Endophenotypes Identifies Potential Therapeutic Targets for Modifying Disease Trajectory

**DOI:** 10.1002/alz.095746

**Published:** 2025-01-09

**Authors:** Thea Jacobson Rosewood, Kwangsik Nho, Shannon L. Risacher, Shiwei Liu, Sujuan Gao, Andrew J. Saykin

**Affiliations:** ^1^ Indiana Alzheimer’s Disease Research Center, Indianapolis, IN USA; ^2^ Indiana University School of Medicine, Indianapolis, IN USA; ^3^ Department of Medical and Molecular Genetics, Indianapolis, IN USA; ^4^ Department of Radiology and Imaging Sciences, IUSM, Indianapolis, IN USA; ^5^ Department of Radiology and Imaging Sciences, Indiana University School of Medicine, Indianapolis, IN USA; ^6^ Indiana University School of Informatics and Computing, Indianpolis, IN USA; ^7^ Indiana University Center for Aging Research, Indianapolis, IN USA; ^8^ Department of Radiology and Imaging Sciences, Center for Neuroimaging, School of Medicine, Indiana University, Indianapolis, IN USA; ^9^ Department of Medical and Molecular Genetics, Indiana University School of Medicine, Indianapolis, IN USA

## Abstract

**Background:**

Alzheimer’s disease (AD) is characterized by longitudinal changes of biomarker endophenotypes over the course of the disease prodrome, onset, and progression. The genetic pathways that influence these heterogenous changes in longitudinal endophenotype trajectories may provide insight into disease mechanisms and represent potential therapeutic targets.

**Methods:**

Longitudinal endophenotypes from the Alzheimer’s Disease Neuroimaging Initiative (ADNI) were selected: amyloid‐β (Amyloid PET and CSF), total tau and phosphorylated tau (CSF), glucose metabolism (FDG PET), neurodegeneration (atrophy on MRI), and cognition (composite scores for memory and executive functioning). Genome‐wide association analysis for the selected longitudinal endophenotypes was performed using Linear Mixed Modelling (LMM; LME4 R package), with (Time x Subject) as a random effect and age as the time variable. Gene‐based association analysis was performed using MAGMA on SNP *P* values from the LMM. The SNP to gene assignment was performed in two steps to select SNPs with a functional relation to each target gene: SNPs within gene transcription start and end positions, and SNPs that have significant eQTLs in brain tissue from the MetaBrain eQTL project. Gene‐based analysis results were then processed for gene‐set enrichment with MAGMA and the C2 curated gene set collection from the Gene Set Enrichment Analysis (GSEA) Molecular Signatures Database (MSigDB).

**Results:**

Pathway enrichment analysis identified 19 pathways (Figure 1) as significantly associated with longitudinal trajectories of AD endophenotypes. These pathways fall into six groups, with each pathway group having stronger association with different types of endophenotypes. Immune and cytoskeletal pathways largely associated with changes in amyloid trajectory. Metabolic pathways associated strongly with changes in amyloid and tau trajectories. Glycosylation pathways were associated with changes in brain atrophy. Pathways related to cell and neuronal signaling associated with changes in cognition, tau, and amyloid trajectories. Cell growth and survival was associated with changes in neurodegeneration trajectory (structural atrophy and hypometabolism).

**Conclusions:**

Pathway enrichment analysis of genetic variation associated with longitudinal changes of AD endophenotypes identified pathways that uniquely associate with trajectories of key AD biomarkers and cognition. These pathways may provide insight into AD pathological mechanisms and constitute new potential therapeutic targets to modify disease trajectory.